# Supplementary material for: The p97/VCP adaptor UBXD1 drives AAA+ remodeling and ring opening through multi-domain tethered interactions
Source: Nat Struct Mol Biol. 2023 Nov 9;30(12):2009–19. doi: 10.1038/s41594-023-01126-0 (PMC10716044; doi:10.1038/s41594-023-01126-0)

Source Data for Extended Data Fig. 1b | Uncropped gels.

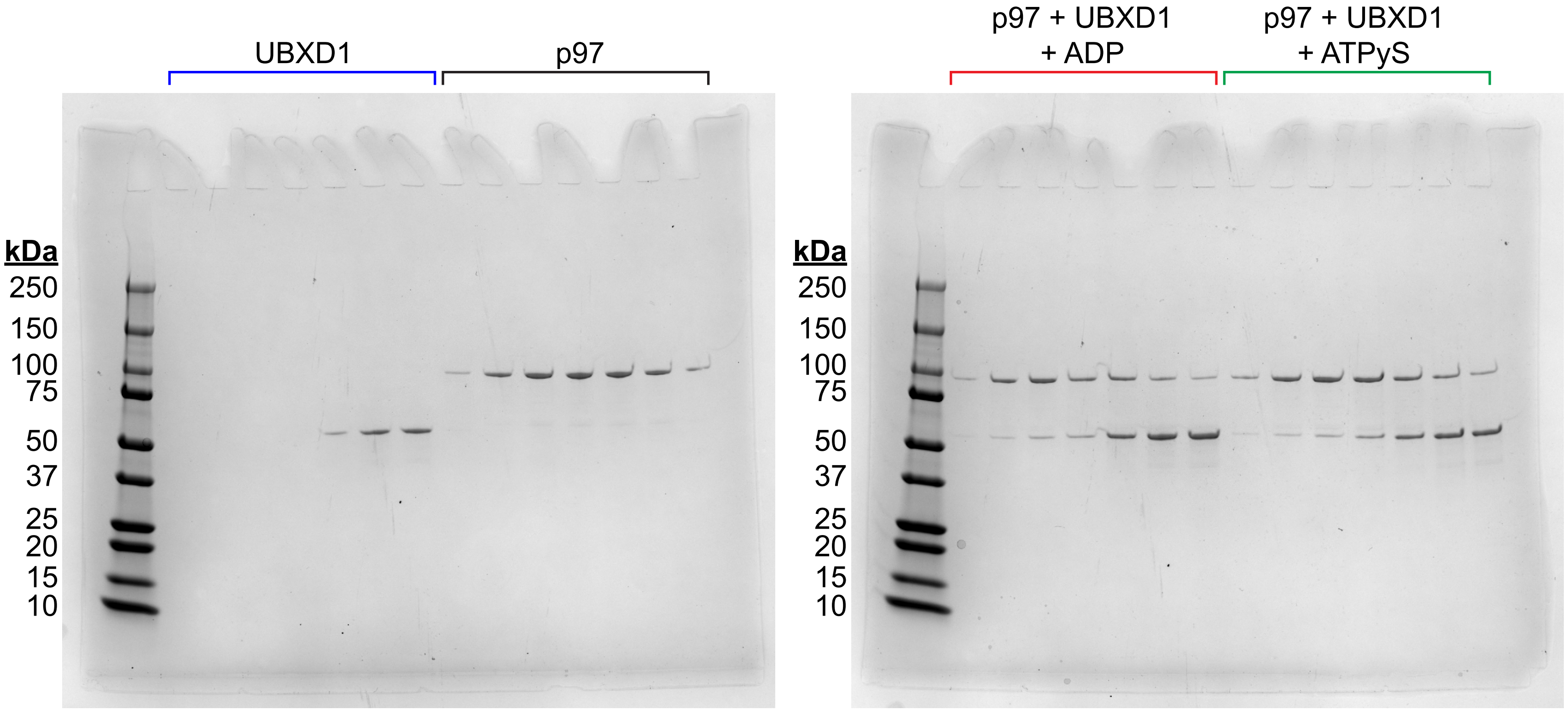

Source Data for Extended Data Fig. 9c | Uncropped gels.

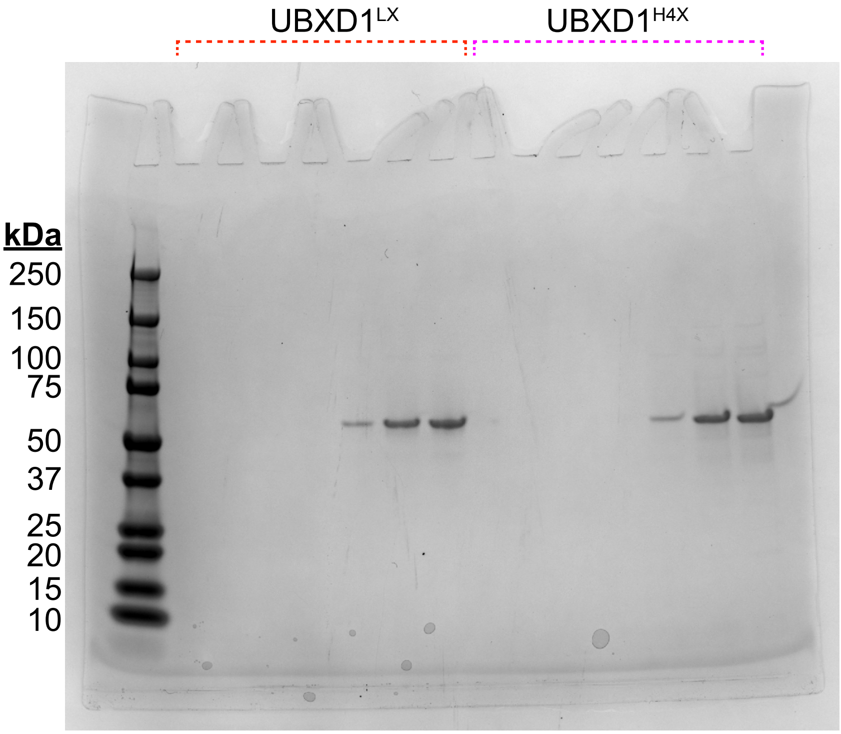

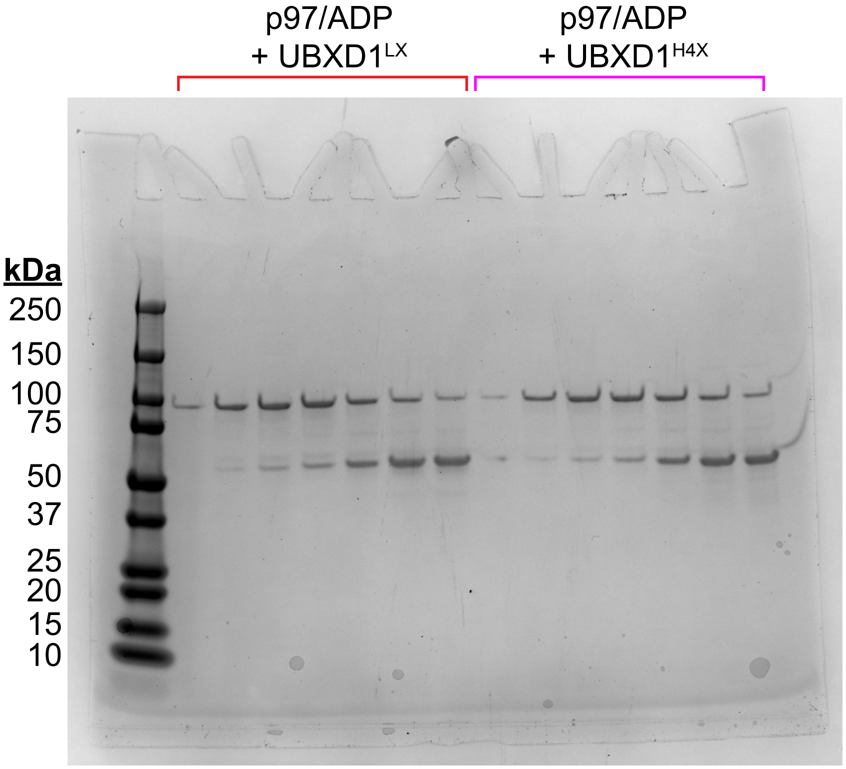

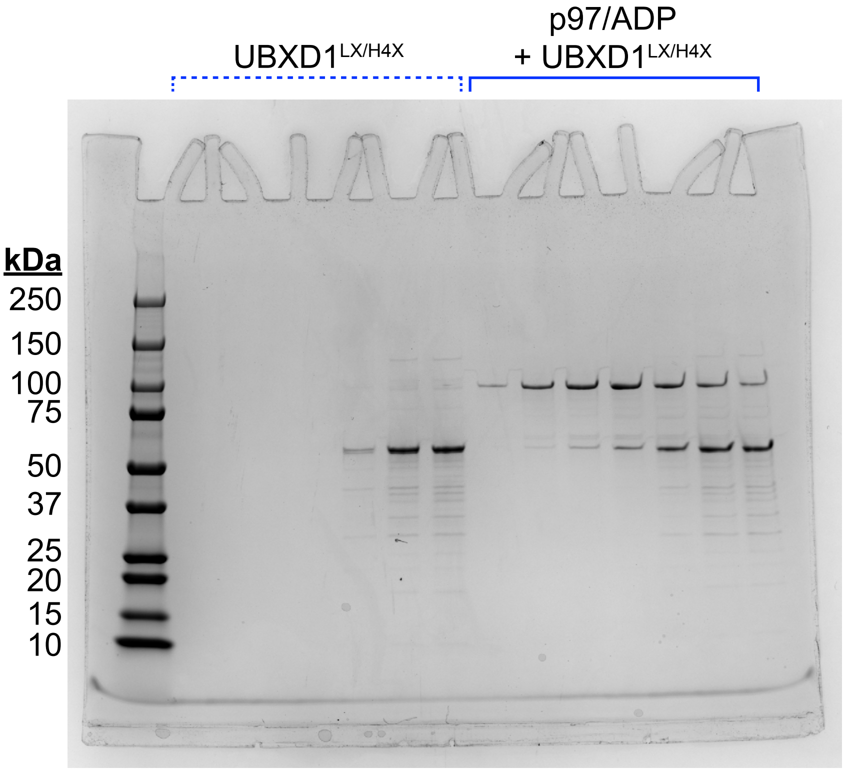

Supplement: Supplementary file 11 — Uncropped gels. [file 41594_2023_1126_MOESM11_ESM.pdf]
